# Practical Results of Higher Education

**Published:** 1899-09

**Authors:** 


ARTICLE III.

### PRACTICAL RESULTS OF HIGHER EDUCATION.

The problems in education will never, apparently cease to be a source of anxiety to those engaged in training young men for service in the world. The effort to prove that the thoroughly educated man is the best man for all business and other relations has been continuous for years, while the opposite view is stoutly maintained that a theoretical education will disqualify for practical service in proportion to its extent and thoroughness.

That there is much truth on both sides of this much mooted question must be admitted. The thorough mechanic may be developed from the man who has devoted his years to the study of the classics, while it is possible to make a learned man from the blacksmith at the forge, a fact frequently demonstrated. The result depends entirely upon the taste of the individual. The scholar may be a mechanic by inclination and habit of thought. Once started in this direction, he will not only make a good mechanic, but will broaden the entire work through the intelligence acquired in other directions. This is equally true of the mechanic. The surgeon may be learned in medical science, but inability to use the instruments required results badly. It is frequently painful to witness the attempt to perform an operation by one who has never acquired skill in this direction.

While it is true, on both sides of the argument, that here and there exceptional cases pass over the boarderland into the domain of the other, it yet remains a fact that the higher the training the lower the mechanical ability. This apparent paradoxical condition of things will, probably, not be admitted by those who have to deal with dental education, but it has become a serious fact in dental teaching.

The cry for years has been for a higher standard. Dentists of the present and future must possess not only a preliminary education of a very high grade, but they must during the college term be trained in all the collateral branches supposed to be of special value to the medically educated student. This has grown year by year until the dental student has been loaded down with an amount of scientific training that absorbs nearly all the three years devoted to the study of dentistry. It is plain that he cannot accomplish two extremes, and, as usually happens, he drops somewhere between, being neither a dentist nor a mechanic.

The writer has been impressed with the fact for years that dentistry, upon its practical side, has been languishing through higher training. The dentists of to-day are not, as a rule, equal as operators or mechanics to those graduated when two years was the maximum limit of school work, while they are measureably superior in all that appertains to higher scientific culture.

The work of filling teeth is not by the majority as dexterously performed as it was twenty years ago. Then good operations were the rule, now they are the exception. Forty years ago dentists were expected to be able to manufacture metal plates with the skill of the jeweler. Now both operator and plate-worker are to a great degree, lost into the higher training; and unless measures be taken to effect an equilibrum between the theoretical and practical, it will not be a generation before the good name of American dentists will be measurably lost in both branches.

Is there any remedy for this state of things? Can the practical and most important part of dental training be conserved and the other, the cultured, be maintained and possibly increased?

It must be apparent to all dental educators that a remedy for this must be found, and that speedily. The students of our best dental colleges are overworked mentally and physically. The curriculum is crowded with subjects. The—so-called—technic training absorbs altogether too much time in the freshman year. A limited amount of this is essential, but the student, at the earliest period, should be placed at the threshold of his serious work in life, upon the the living subject.

The Technic Association recently met in Cincinnati and spent two days discussing these preliminary problems. While this may be well, and the time and money expended not lost, yet it does seem to the writer that there is altogether too much of this kind of work required in the schools.

If this technical work is to be continued and extended, then we may look forward, not to a four years' course, but to a five or even six. There must be a limit found somewhere to this overloading.

The work, as now performed, would, probably not be a serious drawback to acquirement of skill were it properly systematized, but this cannot be done in dental schools as now managed, for the teaching there is not done by men trained for the special purpose of teaching, but by persons taken from the ranks of practitioners, whose livelihood is dependent, not on the emoluments derived from teaching, but from practice. This divided loyalty produces the legitimate result,—imperfect work in the, to them, least important duty.

There is another point worthy the earnest consideration of dental educators. Many of the dental colleges have selected for the most responsible position, that of Dean, a practitioner of medicine. However worthy and skillful in his own line of work, he is not fitted, by training or inclination, for that duty. The result is a lamentable weakness everywhere. This selection is in direct opposition to all experience. The large mechanical establishments would treat with ridicule the proposition made to place at the head of any of these, as general manager, a man trained in law, theology, or medicine, yet this is exactly what is being done in many of our dental colleges. A dental college is a large manufacturing establishment, and to conduct it rightly it must be managed by men trained in that direction and upon strict business principles, or it will be a failure.

Is there any remedy for the condition that confronts dental educators to-day? The answer to this must be found somewhere and somehow. In the opinion of the writer the solution of the problem must be in an entire re-organization of methods.

Pedagogy is a recognized science, and must be applied here as elsewhere. There is a class of mind in every circle that tends in certain directions. It is this variety of intellect that gives force to all the occupants of life. Some will incline to mechanics, others to the purely professional, some find pleasure in teaching. In dental training the latter are indispensible, but as affairs are at present managed these are practically lost.

The first duty of those engaged in dental education would seem to be to select young men exhibiting this talent, and train them for the work. Place them first in subordinate positions, and as they show ability in imparting knowledge make their positions permanent at a continually increasing salary, so that all temptation to practice may be eliminated. In other words, make the position one of life interest, as in other important occupations.

Then systematize the whole method of teaching. Give the student elementary technics, but as early as possible place him on the living subject. Utilize every hour in the day, that no time be lost. Do away with all useless modeling in clay, or carving in other material, making drawings of teeth on paper, or measuring angles of excavators and pluggers, and, in the place of these time-wasteful methods, add on the higher branches, and extend these to the fullest extent compatible with time and the strength of the student. Make the course four years of nine months each of continuous labor.

It may be said that this is a Utopian idea and can never be carried out. It is to the writer a very simple problem. It will, of course, mean the destruction of many dental colleges, for it is understood that there are many schools in small places that would find it impossible to meet these demands, but, on the other hand, there are colleges now established that cold do this and could well afford to make the experiment. The question must be met, for the schools at present are drifting along with haphazard methods that were sufficient for the requirements of the past, but are wholly unsuited to this age, when the demand is for cultivated minds combined with manual dexterity.

Dentistry needs all the intelligence that it is possible to acquire, and it would be folly to eradicate the higher mental development, as it would be equally disastrous to eliminate practical skill. Both must go together, and that college will serve the profession best that can demonstrate the way in which both can be conserved to the advantage of dentistry and to the benefit of humanity.—*International Dental Journal.*

